# Editorial: IPEX 2020: An Expanding Disease Spectrum and Novel Precision Therapies

**DOI:** 10.3389/fped.2022.856920

**Published:** 2022-02-22

**Authors:** Rosa Bacchetta, Talal Chatila

**Affiliations:** ^1^Division of Hematology, Oncology, Stem Cell Transplantation and Regenerative Medicine, Department of Pediatrics, Stanford University School of Medicine, Stanford, CA, United States; ^2^Center for Definitive and Curative Medicine (CDCM), Stanford University School of Medicine, Stanford, CA, United States; ^3^Division of Immunology, Department of Pediatrics, Boston Children's Hospital and Harvard Medical School, Boston, MA, United States

**Keywords:** FOXP3, regulatory T cells, IPEX, autoimmune enteropathy, TSDR

Forkhead box protein 3 (FOXP3) is an essential transcription factor that regulates the functional maintenance of self-tolerance and prevention of autoimmune diseases conferred by CD4+ T regulatory cells (Tregs). The importance of FOXP3 protein in the homeostasis of the immune system is exemplified by *FOXP3* mutations that impair its expression or function and result in an early-onset and life-threatening autoimmune disease: immune dysregulation, polyendocrinopathy, and enteropathy, X-linked (IPEX) syndrome. Since its first recognition in the early 2000's, IPEX syndrome has been a prototype autoimmune monogenic disease, primarily affecting regulatory T-cell mediated immune regulation. In turn, IPEX has revealed key differences between human and mouse FOXP3, thus posing unique challenges for diagnosis and treatment. Importantly, for most patients, IPEX remains a life-threatening and incurable disease.

In this Research Topic experts in the field have shared their contributions, providing updates on our current understanding of IPEX pathogenesis, diagnosis and clinical manifestations of the disease. Although still rare, the increasing awareness of the disease has expanded IPEX diagnosis to older children through the identification of *FOXP3* mutations in patients who present with immune dysregulations as underlying chronic or recurrent gut disease, subtle kidney pathology, skin diseases, recurrent cytopenia or Type 1 diabetes. Therefore, while severe “typical” early-onset IPEX remains the paradigmatic presentation of FOXP3 deficiency, the atypical forms of the disease, comprising late-onset, mild-IPEX or unusual, sometime monosymptomatic disease, are increasingly being observed. This new clinical heterogeneity of IPEX is nicely reviewed in Consonni et al., who summarize reported late-onset and mild forms of IPEX, as well as cases without enteropathies and with unexpected presentations. The authors provide an in-depth update of the genotype phenotype correlation, although the mechanistic impact of *FOXP3* mutations remains largely unexplored. The functional impact of certain mutations located in non-coding and coding regions of the *FOXP3* gene can be predictive of similar phenotypic outcomes. In contrast, several other *FOXP3* mutations, including those in the leucine-zipper or forkhead (FKH) DNA-binding domain, cause variable unpredictable phenotypes in different patients. Moreover, unusual manifestations such as nephropathy or hypogammaglobulinemia are repetitively related to specific *FOXP3* mutations in different patients. Curiously, a number of patients have *FOXP3* mutations, but no clinical manifestations thus requiring no treatment; this cohort confirms the need for more informative genotype-phenotype diagnostic tools that can accurately predict disease severity. Barzaghi and Passerini present a comprehensive summary of the immunological alterations that could provide a more informed IPEX diagnosis when *FOXP3* mutations are detected. These analyses comprise several important tests, such as quantification of the status of the FOXP3-specific epigenetic marker (TSDR) or assessing Treg-cell functional suppression, that are currently performed on a research basis in specialized centers. Our current knowledge of the pathogenesis of IPEX exemplifies the broader role of FOXP3 in causing immune dysregulation, and the direct effect of dysfunctional Treg cells. *FOXP3* mutation in CD4^+^ T cells can also impact expression of the various FOXP3 isoforms revealing both a fundamental difference between the human and mouse protein and an additional diagnostic challenge. Indeed, as reviewed by Mailer, human, but not murine, FOXP3 is expressed as three major alternatively spliced isoforms. Notably, alterations in the relative expression of each isoform have been observed in immunological abnormalities leading to autoimmunity or inflammation, thus adding a potential level of complexity to the pathogenic impact of *FOXP3* mutations in IPEX. Although further studies are required to assess the consequences of different *FOXP3* mutations in altering FOXP3 isoform expression, and indeed to fully determine the functions of the isoforms themselves, the importance of maintaining expression of each is clear, and should be taken into consideration in developing new gene therapy approaches for IPEX. Lastly, a fascinating examination of the epigenetic and posttranscriptional regulation of FOXP3 is discussed by Grover et al., with findings supporting the development of a new potential therapeutic intervention to treat IPEX.

In summary, studies of FOXP3 in IPEX have shown that altered genetic composition, biochemical features, epigenetic, and posttranscriptional regulation can cause a wide spectrum of disease manifestations ranging from no obvious impairments to the life-threatening early-onset form of IPEX that is the hallmark of diseases resulting from ineffective immune regulation. Intriguingly, in its worst form, IPEX can manifest *in utero*, as uniquely demonstrated by Carneiro-Sampaio et al. These observations begin to clarify the primary role of immune regulation at the molecular and system level in the developing embryo, and stimulate new questions about the origin of autoimmunity and its interrelationship with fetal cell and tissue development.

Despite much progress and some success treating with hematopoietic stem cell transplantation, IPEX remains an incurable disease. The updated concepts summarized in this Research Topic will hopefully stem new targeted precision medicine treatments and inspire new gene therapy approaches that could in “one shot” reverse this disease.

## Author Contributions

All authors listed have made a substantial, direct, and intellectual contribution to the work and approved it for publication.

## Conflict of Interest

The authors declare that the research was conducted in the absence of any commercial or financial relationships that could be construed as a potential conflict of interest.

## Publisher's Note

All claims expressed in this article are solely those of the authors and do not necessarily represent those of their affiliated organizations, or those of the publisher, the editors and the reviewers. Any product that may be evaluated in this article, or claim that may be made by its manufacturer, is not guaranteed or endorsed by the publisher.

